# Habitat with small inter-structural spaces promotes mussel survival and reef generation

**DOI:** 10.1007/s00227-018-3426-8

**Published:** 2018-10-04

**Authors:** Camilla Bertolini, W. I. Montgomery, Nessa E. O’Connor

**Affiliations:** 1 School of Biological Sciences, Queen’s University of Belfast, 97 Lisburn Road, Belfast, BT9 7BL Northern Ireland, UK; 2 NIOZ Royal Netherlands Institute for Sea Research, Department of Estuarine and Delta Systems, Utrecht University, PO Box 140, 4401 NT Yerseke, The Netherlands; 3 School of Natural Sciences, Zoology Building, Trinity College Dublin, Dublin 2, Ireland

## Abstract

Spatially complex habitats provide refuge for prey and mediate many predator–prey interactions. Increasing anthropogenic pressures are eroding such habitats, reducing their complexity and potentially altering ecosystem stability on a global scale. Yet, we have only a rudimentary understanding of how structurally complex habitats create ecological refuges for most ecosystems. Better informed management decisions require an understanding of the mechanisms underpinning the provision of physical refuge and this may be linked to prey size, predator size and predator identity in priority habitats. We tested each of these factors empirically in a model biogenic reef system. Specifically, we tested whether mortality rates of blue mussels (*Mytilus edulis*) of different sizes differed among: (i) different forms of reef structural distribution (represented as ‘clumped’, ‘patchy’ and ‘sparse’); (ii) predator species identity (shore crab, *Carcinus maenas* and starfish*, Asterias rubens*); and (iii) predator size. The survival rate of small mussels was greatest in the clumped experimental habitat and larger predators generally consumed more prey regardless of the structural organisation of treatment. Small mussels were protected from larger *A. rubens* but not from larger *C. maenas* in the clumped habitats. The distribution pattern of structural objects, therefore, may be considered a useful proxy for reef complexity when assessing predator–prey interactions, and optimal organisations should be considered based on both prey and predator sizes. These findings are essential to understand ecological processes underpinning predation rates in structurally complex habitats and to inform future restoration and ecological engineering practices.

## Introduction

Habitat complexity plays a key role in mediating biotic interactions, such as predator–prey relationships (Heck and Crowder 1991; Warfe and Barmuta 2004; Klecka and Boukal 2014). Structurally complex habitats may provide refuge space for prey (Křivan 1998; O’Connor and Crowe 2008), thus modifying predator–prey dynamics (Beck 1995; Barrios-O’Neill et al. 2015), and lead to a cascade of indirect effects on multiple trophic levels (Grabowski and Kimbro 2005; Grabowski et al. 2008; O’Connor et al. 2013). Spatial refuges within complex habitats can be of particular importance for smaller individuals (Hacker and Steneck 1990; Strain et al. 2017), including recent recruits and juveniles, which are usually more vulnerable to predation than larger individuals (Gosselin and Chia 1995). Studies of habitat complexity often use different definitions of complexity or confound complexity with other habitat characteristics, such as surface area or heterogeneity (Beck 2000; Frost et al. 2005; Kovalenko et al. 2012; Loke et al. 2015), which can lead to misuses of these metrics for management purposes (Wedding et al. 2011). Habitat complexity per se is often used as an over-arching term that encompasses variation in several habitat ‘components’, e.g. density of specific habitat component such as pits, pneumatophores or crevices (McCoy and Bell 1991), which limits the application of the results of studies using generic or obtuse terminology. A more useful approach is to use only specific metrics of individual habitat components (Beck 2000).

Many structurally important habitats, e.g. rainforests, saltmarshes, and aquatic biogenic reefs, are under threat from anthropogenic disturbances (Ellison et al. 2005; Airoldi et al. 2008; Silliman et al. 2009; Newbold et al. 2014; Firth et al. 2015). Biogenic reefs formed by bivalves play an essential role as ecosystem engineers (Geraldi et al. 2017) by: (i) promoting higher levels of biodiversity than surrounding local environments (Gutierrez et al. 2003; O’Connor and Crowe 2007); (ii) providing habitat that acts as a nursery for commercially important species (Kent et al. 2016, 2017); (iii) stabilising sediments (Meadows et al. 1998); (iv) acting as natural wave barriers and protecting soft coastal habitat (Stone et al. 2005); and (v) contributing substantially to nutrient cycling (Kellogg et al. 2013). The loss of such biogenic habitat following disturbance events can lead to changes in many biotic interactions, which can impede the recovery of a system following further disturbances (Lotze et al. 2006; Bertness et al. 2015; Mrowicki et al. 2016). It is often assumed that biogenic reefs have a self-sustaining mechanism, such that once a reef is established, its complex structure provides refuge from predation, which facilitates recruitment (Bertness and Grosholz 1985; Nestlerode et al. 2007; Walles et al. 2015, 2016) and maintains a healthy and stable reef system. When a reef is damaged, however, this process will be diminished (Lenihan 1999), which could de-stabilise a reef-dominated system by reducing the establishment of new recruits and subsequent reef re-formation (Barrios-O’Neill et al. 2017; Fariñas-Franco et al. 2018; Fariñas‐Franco and Roberts 2018), potentially leading to an alternative stable state which may be represented as a ‘degraded’ system lacking in complexity (Petraitis and Dudgeon 2004).

It is essential to understand how predator–prey relationships interact with spatial complexity (Warfe et al. 2008; Hesterberg et al. 2017) so that we can comprehend how these processes are linked to refuge availability, which underpins biogenic reef formation and persistence. The density of reef-forming species can be used as a proxy to estimate the organisation (or spatial arrangement) of vertical objects in horizontal space (Bell et al. 1991). Density is a tractable measure that can be quantified and manipulated experimentally and, thus provides useful insights into the recruitment dynamics of reef-forming species (Carroll et al. 2015). The density of reef-formers, however, is often confounded with other factors, such as volume or abundance of individuals (e.g. Humphries et al. 2011a, b). The aim of our study was to test whether different, but typical, spatial arrangements of habitat structure within an experimental reef system affected the survival of small mussels. Specifically, by ensuring that other components of habitat structure were constant and manipulating only density, we tested directly whether the size of interstitial spaces available affected overall mussel survival, and whether different spatial arrangements of this habitat provided better protection for small-sized mussels from differently sized predators (Toscano and Griffen 2014; Bartholomew et al. 2016). Additionally, we tested whether refuge efficacy differed between species of common predators with distinct methods of catching and killing prey (O’Connor et al. 2008; Farina et al. 2014). Small interstitial spaces which are typical of mussel beds may be beneficial for the smaller mussels, whilst being of limited use for larger mussels, which may become more vulnerable to predation from larger or different predator species (Enderlein et al. 2003; Calderwood et al. 2015b).

**Fig. 1:**
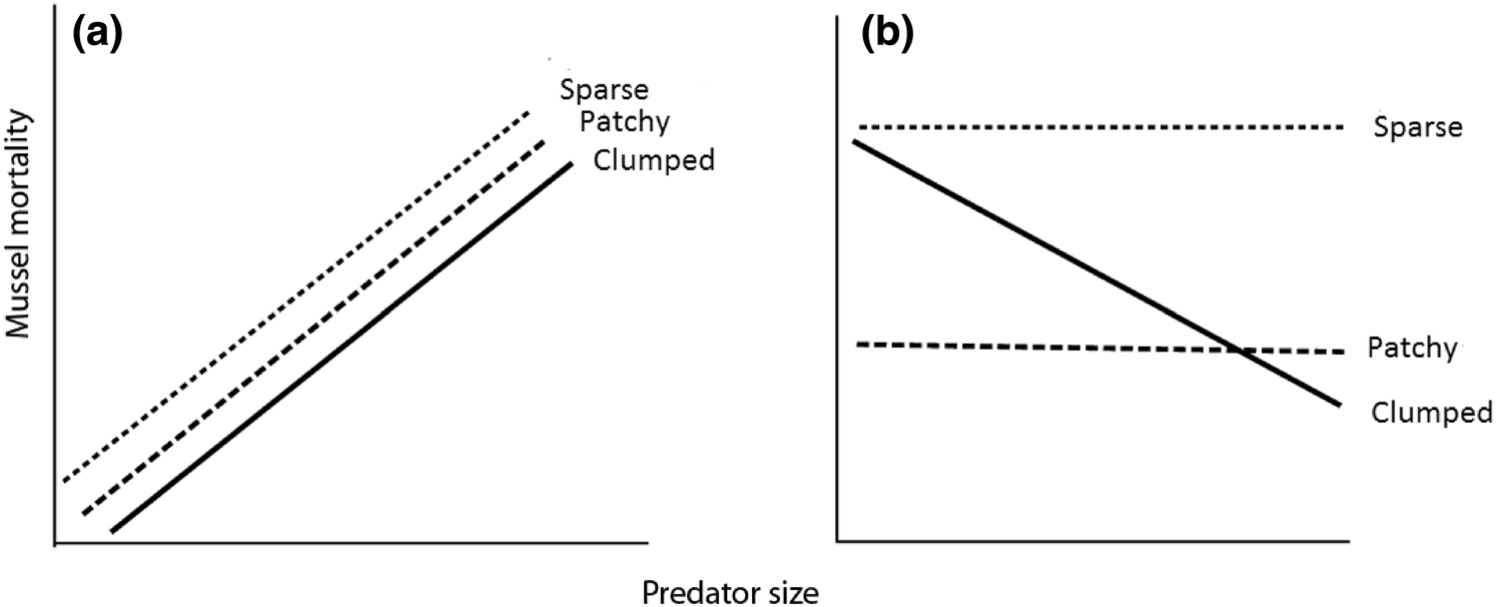
Hypothetically predicted predator–prey relationships in ‘sparse density’ (dotted line), ‘patchy organisation’ (dashed line) and ‘clumped density’ (black line) treatments with predators of increasing size for: **a** total mussel mortality, and **b** small mussels

In two separate experiments, the effects of predation of two common benthic predators (the shore crab, *Carcinus maenas,* and the starfish, *Asterias rubens*), on their shared prey (mussels) was quantified using artificial reefs that were designed to represent three different forms of habitat organisation. The size of the predators and of prey was also manipulated to test explicitly for size-dependent effects and to identify mechanisms that underpin predation in this system. Specifically, both experiments tested the hypotheses that: predation rates on mussels are dependent upon habitat organisation (‘sparse’, ‘patchy’ and ‘clumped’) with (1) mortality rate of small mussels being lowest in the ‘clumped’ habitat organisation; whereas (2) a ‘patchy organisation’, with heterogeneous sizes of refugia available, will provide generally more options for refuge, thus decreasing the mortality of larger individuals; and that (3) the size of predators will affect the mortality rates of their prey, in relation to accessibility to the interstitial spaces (Fig. 1), while (4) the ratio of small mussel mortality compared to total mortality will only differ with habitat spatial organisation.

## Materials and methods

### Experimental design

The experiments were conducted in outdoor, flow-through mesocosms at Queen’s University Marine Laboratory, Portaferry, Northern Ireland. Mesocosms consisted of opaque plastic boxes (55.5 × 35.5 × 22 cm) arranged on shallow tables and supplied independently with sand-filtered, seawater from the adjacent Strangford Lough cascading from the top on all of the tanks (further detail in Mrowicki and O’Connor 2015).

The first experiment quantified predation rates of crabs on mussels based on: (i) habitat organisation as a fixed factor (with three levels: ‘sparse’, ‘patchy’, and ‘clumped’); (ii) crab size (as a continuous variable); and (iii) trial (3 levels), which was included as a random factor. The second experiment, quantified predation rates of starfish on mussel beds based on: (i) habitat organisation as a fixed factor (with three levels: ‘sparse’, ‘patchy’, and ‘clumped’); (ii) starfish size as a fixed factor (with two levels: small, large) given the lack of intermediate-sized individuals; and, (iii) trial (2 levels), which was included as a random factor.

**Fig. 2:**
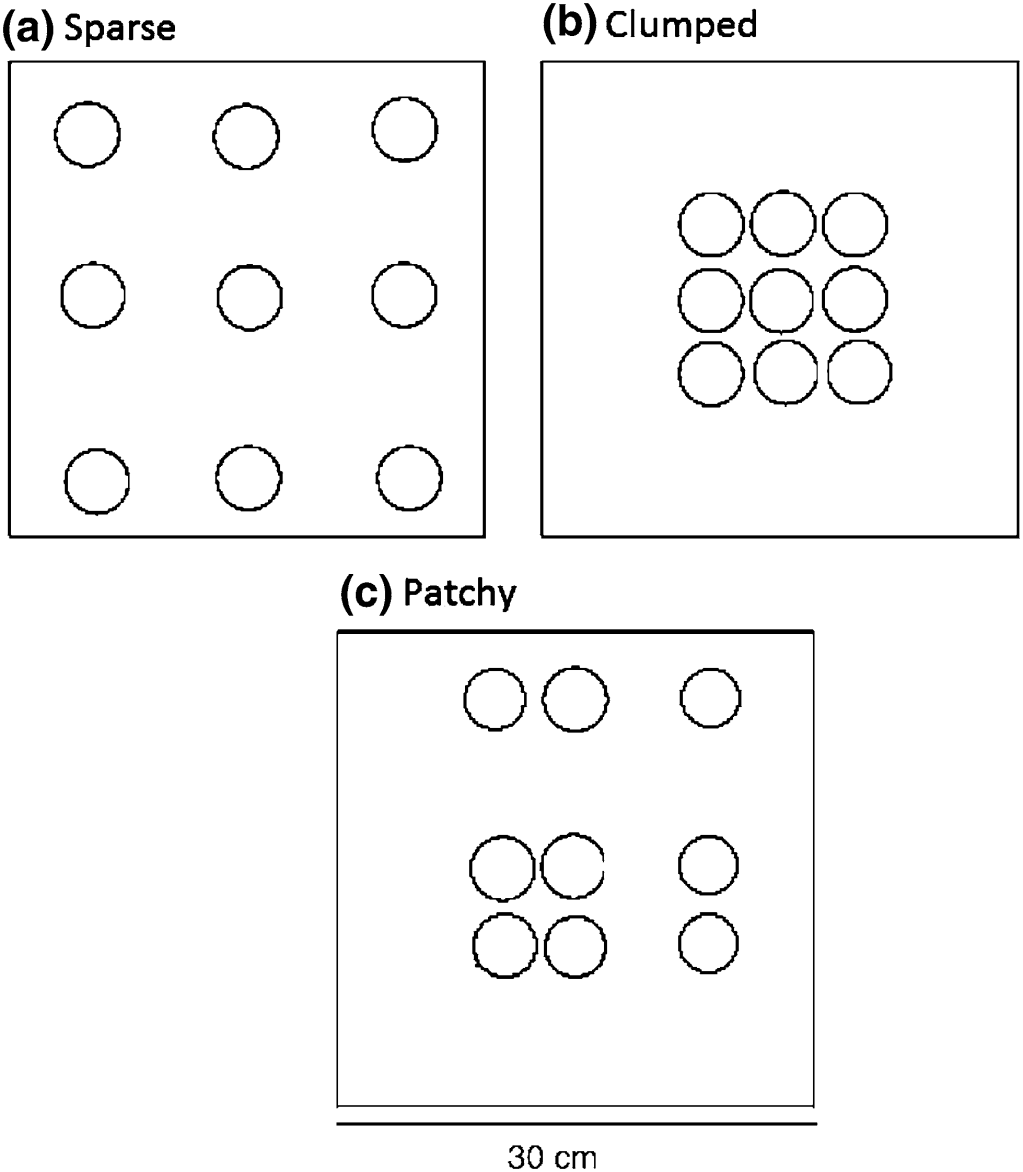
Top view (dorsal) of the design of artificial reefs with three different spatial organisations (experimental treatments). **a** ‘Sparse’, **b** ‘clumped’, **c** ‘patchy’. Squares represent perspex tile, circles represent PVC pipe

Artificial reefs were designed to manipulate interstitial space size whilst keeping overall area constant in all treatments. Artificial reefs were constructed from 30 × 30 cm Perspex plates (Fig. 2) each containing 9 mimics made from white PVC pipe (diameter 2.5 cm, height 3.5 cm). The experimental treatments were designed to represent reefs with ‘sparse’, ‘patchy’ and ‘clumped’ distributions based observations of local mussel abundance patterns. For example, in the sparse distribution treatment, individual mussel mimics were each placed 8.5 cm apart, which is representative of a degraded habitat of isolated mussels (Fariñas-Franco et al. 2014). This sparse treatment was hypothesised to provide limited or no physical refuge available. In the patchy distribution treatment, three mussel mimics were placed 8.5 cm apart, another three were 3.5 cm apart and another three were 1 cm apart, representing a patchy mussel organisation. This patchy treatment was hypothesised to provide several refugia for mussels of various sizes. In the clumped distribution treatment, the distance between all nine mussel mimics per plate was 1 cm, representing a densely packed and uniformly distributed mussel reef. This clumped treatment was hypothesised to provide refuges for small mussels.

### Experiment 1: *Carcinus maenas* predation

This experiment ran from December 2015 to January 2016 with three trials comprising all treatments running over this time period. Each of the three experimental treatment (sparse, patchy and clumped) were replicated eight times in each trial yielding a total of 72 experimental units. New crabs and mussels were collected for each experimental trial.

Mussel sizes used in the experiment were designed to mimic two common sizes available on mussel patches at local intertidal, soft-sediments shores: one small and able to find refuge and a larger one which would not be able to hide in small interstitial spaces. The specific abundance of the two classes was chosen to mimic natural conditions (mean abundance ± SE: small mussels 6.4 ± 1, large 4.8 ± 1.2/900 cm^2^), thus ten mussels (five mussels of two size classes, mean shell length ± SE: small, 11.2 ± 0.4 mm and large 22.7 ± 0.5 mm) were added to each experimental plate described above, which was then allocated randomly to a mesocosm. All mussels selected had clean shells, were collected from a local shore (54°29′15.96″N, 5°32′24.74″W) and were within the recorded feeding range of *C. maenas* (Mascaró and Seed 2000; Enderlein et al. 2003; Calderwood et al. 2016). Following pilot studies, mussels were allowed to acclimatise for 24 h prior to the addition of the predator (*C. maenas*), allowing them to find a suitable space within the model reef and to form a byssal attachment to the structure. All mussels attached to the habitat provided by the artificial structures, but no mussel-to-mussel clumping was observed during the experiment.

Shore crabs, *C. maenas,* were collected from a local shore (54°23′26.6424″N, 5°34′19.5126″W) and to standardise motivation to feed, only male individuals without any visible signs of damage were selected for use in this study. Crab carapaces were measured with digital calipers. Only crabs with carapace width > 30 mm were selected because crabs of this size are known to have *M. edulis* as a large component of their diet (Ropes 1968). All crabs were transferred into three holding tanks (35 L) and acclimatised for 24 h with ad libitum food supply of *M. edulis*. Crabs were then starved for 48 h prior to the beginning of the experiment to standardise hunger level (Elner and Hughes 1978). One crab was assigned randomly to each mesocosm and left to forage overnight for 18 h from 16.00 to 10.00 h (Ropes 1968, Calderwood et al. 2016). At the end of each trial, crabs were removed and the number of surviving small and large mussels was recorded. First, we analysed total mussel mortality and small mussel mortality. Then to assess whether the consumption of small mussels differed among experimental treatments and across the range of predator sizes we further analysed the small mussels consumed as a proportion of all mussels consumed.

### Experiment 2: *Asterias rubens* predation

Two experimental trials were run in February and May 2016. Each of the three experimental treatments (sparse, patchy and clumped) was replicated four times in per each starfish size class per trial yielding a total of 48 experimental units. New starfish and new mussels were collected prior to the start of each experimental trial. To keep mussel density similar to the previous experiment and to avoid confounding effects of clumping while investigating habitat use, nine mussels (three mussels of three size classes: small, mean shell length ± SE 9.5 ± 0.4 mm, medium 20 ± 0.5 mm, and large 32.4 ± 0.6 mm) were added to each plate, which was then randomly allocated to a mesocosm. Clean shelled *M. edulis* were also collected at low tide from a local shore (54°29′15.96″N, 5°32′24.74″W), and sorted into the three size classes: all mussels were chosen to be sizes normally consumed by starfish (Hummel et al. 2011), with the small mussels able to seek refuge in the ‘clumped’ reef organisation. In contrast, medium and large mussels were chosen since they were excluded from the refuge space, with medium-sized mussels accessible by smaller starfish and large mussels possibly chosen by larger starfish (Hummel et al. 2011; Calderwood et al. 2015b). Mussels were then left for 24 h to acclimatise, move to a suitable space and form a byssal attachment to the structure. Again all mussels attached to the habitat provided by the artificial structures, but no mussel-to-mussel clumping was observed during the experiment.

Undamaged starfish were collected manually from a local shallow subtidal shore (54°23′29.9214″N, 5°34′29.301″W). Owing to the starfish sizes available locally, starfish were sorted by size with small (arm length 24.2 ± 1.7 mm) and large (96.02 ± 0.45 mm) individuals transferred into separate holding tanks (volume 330 L) where they were left to acclimatise for 24 h with ad libitum food supply (*M. edulis*). Starfish were then starved for 7 days prior to the beginning of experiment to standardise hunger levels. At the start of each trial, a small or large starfish was assigned randomly to a mesocosm and left to forage for 4 days. At the end of the foraging period, starfish were removed and the number of surviving mussels was recorded. Percentage mortality of all mussels and percentage mortality of smaller mussels was used for statistical analyses. To differentiate whether the consumption of the small class changed with organisation or predator size we further considered the proportion of small mussels consumed in relation to total mortality, expressed as a percentage.

### Statistical analysis

All analyses were carried out using R (R Development Core Team 2015). Data were tested for homogeneity of variances using Levene’s test in the *car* package. For both experiments, linear mixed models fitted by maximum likelihood *t* tests using Satterthwaite (1946) approximations of degrees of freedom were chosen to incorporate the random effect of ‘trial’ (Bates 2005; package *lme4*). The initial model included fixed terms (‘habitat organisation’, ‘predator size’), their interactions and the random factor (‘trial’). Where the interactions were not significant they were removed from the model. The models with and without interactions were then compared based on AIC score and that with a lowest score was chosen. Moreover, the random effect ‘trial’ always explained < 5% of the total variance. Therefore, mixed linear models were compared to linear models that did not include the random factor using AIC scores. AIC were lower for linear models in all cases. The residuals were tested for normality with Shapiro–Wilk test and the model was validated. Type II ANOVA tables using the *Anova* function in the package *car* was then used to generate *p* values and test for significance. If terms were significant, pairwise comparisons between levels were carried out using least means squares estimates based on Tukey adjustments (*lsmeans* and *multcomp* packages).

## Results

### Experiment 1: *Carcinus maeanas* predation

**Fig. 3:**
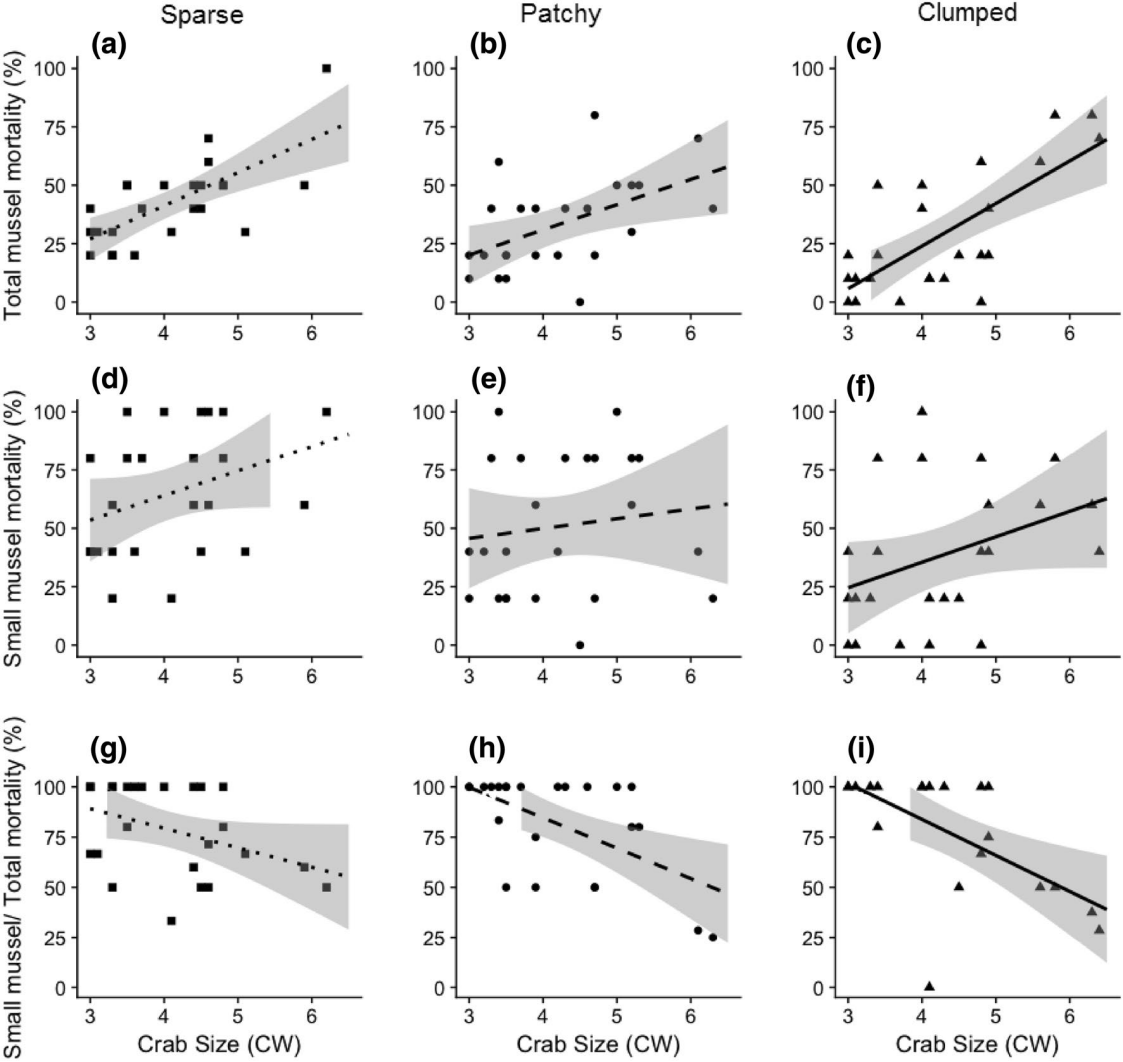
The relationship between mussel mortality and crab size in “sparse density” (squares, dotted line, **a**, **d**, **g**), “patchy” (circles, dashed line, **b**, **e**, **h**) and “clumped density” (triangles, continuous line, **c**, **f**, **i**) organisations based on: total mussel mortality (panels **a**–**c**), small mussel mortality (**d**–**f**), and proportion of small mussels mortality compared to total expressed as percentage (**g**–**i**). Lines and shading represent 95% confidence intervals extrapolated from linear models

Small mussel (ca. 10 mm in shell length) mortality was significantly lower in the ‘clumped density’ treatment (*F*_2,68_ = 5.9, *p* < 0.01, Table 1, Fig. 3d–f). A ‘patchy’ organisation did not contribute to a decreased mortality (*p* > 0.05). The lowest total mussel mortality was in the ‘clumped density’ organisation (*F*_2,68_ = 5.88, *p* < 0.01, Fig. 3a–c, Table 1) and increased with increasing crab size (*F*_1,68_ = 5.9, *p* < 0.001, Fig. 3a–c). There was no interaction between organisation and crab size, however, both small and total mussels mortality was found to increase with increasing size of crabs (*F*_1,68_ = 5.9, *p* < 0.05, Fig. 3d–f). The proportion of small to total mussel mortality did not change with different organisations, however, this ratio decreased with increasing crab sizes (*F*_1,63_ = 25.1, *p* < 0.0001, Fig. 3g–i).

**Table 1:** Pairwise comparisons using least squares means estimates between complexity treatments for all mussel mortality in the presence of a crab

Pairwise	Estimate	Std. error	*t* ratio	*p*
Total mussel mortality				
Sparse-clumped	**16.25**	**4.8**	**3.44**	**0.004**
Sparse-patchy	11.12	4.8	2.29	0.063
Patchy-clumped	5.11	4.8	1.06	0.541
Small mussel mortality				
Sparse-clumped	**28.14**	**8.2**	**3.43**	**0.003**
Sparse-patchy	15.31	8.2	1.87	0.155
Patchy-clumped	12.82	8.2	1.56	0.266

Significant differences in bold

### Experiment 2: *Asterias rubens* predation

**Fig. 4:**
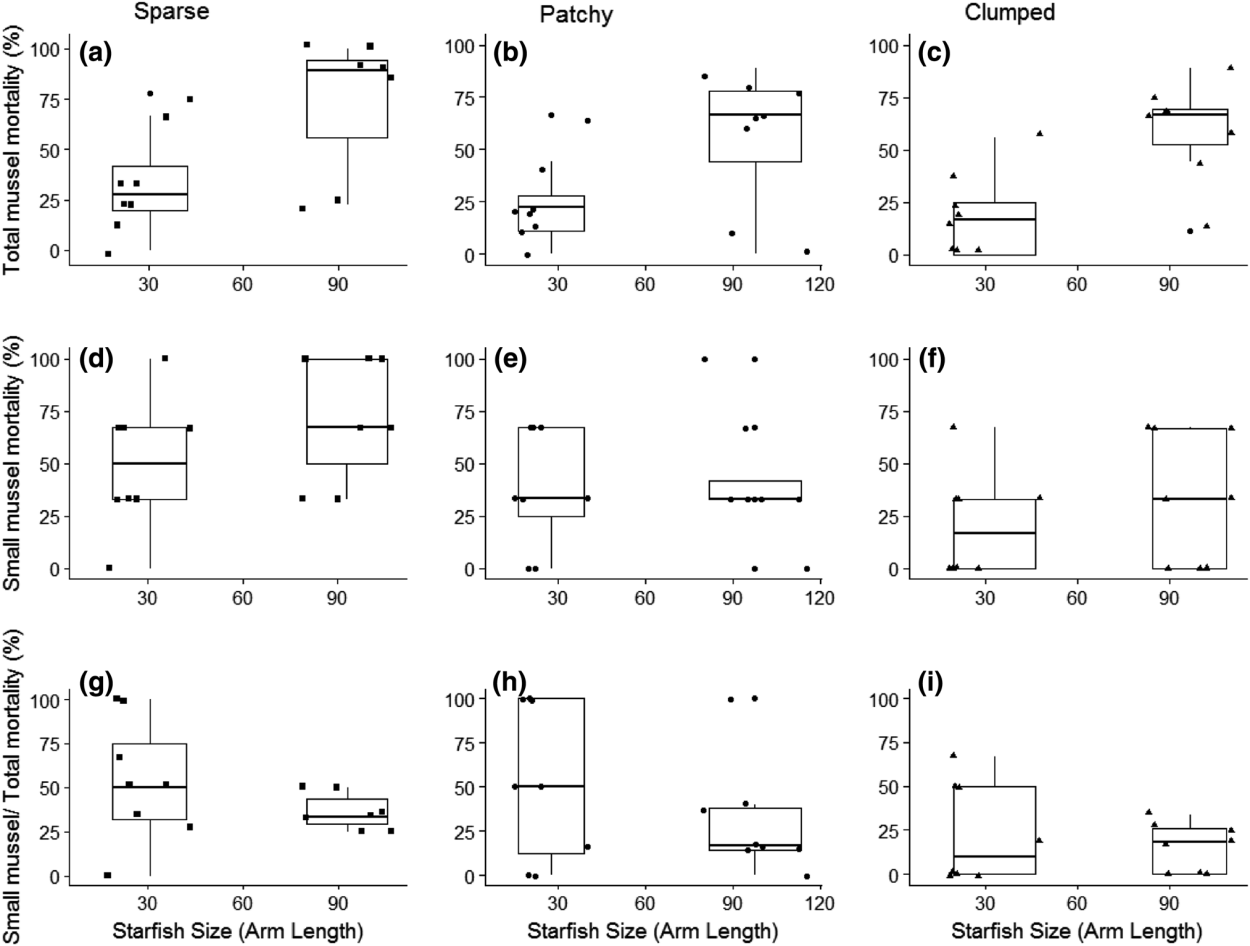
Boxplot with overlying raw data representing medians and interquartile ranges of percentage mortality of mussels exposed to different starfish sizes in “sparse density” (squares, **a**, **d**, **g**), “patchy” (circles, **b**, **e**, **h**) and “clumped density” (triangles, **c**, **f**, **i**) organisations based on: total mussel mortality (**a**–**c**), small mussel mortality (**d**–**f**), and proportion of small mussels mortality compared to total expressed as percentage (**g**–**i**)

Small mussel (ca. 10 mm in shell length) mortality was significantly lower in the ‘clumped density’ (*F*_2,44_ = 6.2, *p* < 0.01, Table 2, Fig. 4d–f), however, this did not differ among starfish sizes, nor were there interactions between habitat organisation and predator size (*p* > 0.05).

**Table 2:** Pairwise comparisons using least squares means estimates between complexity treatments for small mussel mortality in the presence of a starfish

Pairwise	Estimate	Std. error	*t* value	*p*
Sparse-clumped	**35.41**	**10.2**	**3.5**	**0.003**
Sparse-patchy	22.92	10.2	2.2	0.073
Patchy-clumped	12.5	10.2	1.2	0.443

Significant differences in bold

A ‘patchy’ organisation did not contribute to a decreased mortality (*p* > 0.05).

In the starfish experiment (Fig. 4) total mussel mortality rate did not differ among habitat organisations, however, total mussel mortality increased when starfish were larger (*F*_1,44_ = 25.5, *p* < 0.001, Fig. 4a–c). The proportion of small to total mussel mortality decreased with increasing complexity of organisation from sparse to clumped (*F*_2,38_ = 3.5, *p* < 0.05), and was lower when starfish were larger (*F*_1,38_ = 8.8, *p* < 0.001, Fig. 4g–i).

## Discussion

In agreement with our initial hypotheses (Fig. 1), we found that in the presence of crabs, different habitat organisations affected mussel predation rates differently, and that mussel survival was greatest in the clumped density treatment. In contrast, we did not identify any effects of different habitat organisation on total mussel mortality in the presence of starfish. However, when predation effects were examined for small mussels only, clumped habitat organisation significantly increased survival of small mussel classes independent of predator species. There was no effect of organisation on the ratio of small mussels consumed compared to total mortality when crabs were the predators, while there was a slight decrease in this ratio with increasing organisation density when starfish were predators.

In the present study, habitat organisation was manipulated only with regard to horizontal space by manipulating object (mussel mimic) density. Despite this simplification of variability in reef structure, which did not consider variations in three dimensions (Hesterberg et al. 2017), and used habitat mimics, it was a highly suitable proxy to test for effects of different interstitial space sizes among objects (Bartholomew and Burt 2015; Bartholomew et al. 2016) and was a suitable mediator of predation rates. Where habitat organisation was clumped, prey mortality, in general, was lower, suggesting that altering habitat organisation even in two dimensions reveals useful mechanistic insights with regard to refuge availability and refuge efficacy. The reduction in mortality owing to predation in habitats with clumped density organisation could be driven by the ability of prey to hide from predators and/or the inability of predators to reach inside the refuge to access prey (Klecka and Boukal 2014), rendering the task too difficult or energetically prohibitive (Dolmer 1998).

Variability in the efficacy of refugia was prey size specific. Small mussels benefitted from the clumped-density organisation, where they found refuge. Mortality for this size class was lower in the clumped compared to the lower density organisation treatments, suggesting an effect of habitat organisation and not general prey size preference per se. These findings suggest that promoting a habitat with small available refuge spaces can significantly increase survival of small mussels in the presence of predators, and thus enhance their potential to increase reef sustainability and growth (van de Koppel et al. 2005; Commito et al. 2014; Folmer et al. 2014; Bertolini et al. 2017).

The size of the predators affected mussel mortality positively and the lack of interactions showed that this occurred independently of habitat organisation. This result was in contrast to our third and fourth hypotheses, as we found that in general consumption rate, particularly the consumption of larger mussels, increased with predator size. Smaller predators in both experiments were small enough to use the interstitial spaces in the ‘clumped density’ organisation, and were observed doing so. In contrast to predictions, larger crabs were able to feed on small mussels in the clumped organisation. This did not occur when starfish were present because predator size did not affect their predation rates on mussels in the clumped organisation. This could be because of their different physical feeding methods, with crabs having strong claws (Vermeij 1977) and able to reach into a refuge space and pull a mussel out. Starfish might not be able to reach and secure a small mussel in a small space because of the bulk of their arms and relative weakness of a hydrostatic tube foot system leading to a poor capacity to grip prey (Dolmer 1998). Moreover, predators with different prey-detecting strategies (visual vs chemical vs tactile cue) may have different rates of prey encounter in complex habitats (Farina et al. 2014; Klecka and Boukal 2014).

Larger predators consumed more mussels and tended to prefer larger sizes, whilst smaller predators were limited to consumption of smaller mussels, suggesting that larger mussels may be able to escape predation from smaller predators in all of the organisations tested here. This was consistent with results from studies of crab predation on oyster reefs where the presence of large crabs was the major determinant of mussel and oyster mortality (Toscano and Griffen 2012; Pickering et al. 2017). Thus, mussels that are excluded from refuge space and are of edible size may suffer from high mortality rates. While other experiments found that, *A. rubens* (Hummel et al. 2011) and *C. maenas* (Smallegange and Van Der Meer 2003) often prefer small prey items. We found that overall small mussel mortality also increased with increasing predator size, and the ratio of small mussels mortality compared to total mortality decreased with increasing predator size, suggesting that consumption of larger size classes is important for larger predator sizes. In natural reefs, refugia may be highly variable in size with suitable but limited refuge space for all size classes, however, for damaged reefs to recover it is important that refugia for small mussels is present so they can grow to regenerate a self-sustaining reef.

Different predator species were found to have generally similar effects on mortality rates, but some differences were highlighted. For example, larger crabs were found to eat small mussels in the clumped density organisation. This was not observed in trials involving large starfish. The importance of considering predator assemblage composition has been highlighted for commercial mussel seeding operations (Calderwood et al. 2015a) and this is reinforced by the present study. Clumped mussel habitat can also be beneficial for smaller predators which may hide therein (Thiel and Dernedde 1994), highlighting the importance of considering the ontogenetic and behavioural responses of predators (Pirtle et al. 2012). It is known, for example, that mussel reefs are nursery grounds for whelks (Kent et al. 2016) and crabs (Lindsey et al. 2006) and the size of small predators used in this experiment may spend more time sheltering from larger predators in refuge space afforded by a reef than actively feeding. This should be tested empirically.

It is concluded that refuge efficacy in reducing mortality from predation is greatest in clumped density habitat organisation but is strongly dependent on prey size rather than predator size. This contrasts with previous research, which found that space size relative to predator width (Sp/Pr) was one of the most important predictors of survivorship, with prey survivorship decreasing sigmoidally with increasing Sp/Pr (Bartholomew et al. 2000). We recommend that refuge size should be evaluated in relation to prey size (Sp/Py) (Hacker and Steneck 1990; Bartholomew and Shine 2008; Bartholomew 2012), that habitats containing multi-sized interstitial spaces should be promoted to offer refuge to multiple size classes, and ultimately should be context-specific with regards to the identity of predators present in the system.

Our findings demonstrate the use of spatial organisation as a measure of habitat complexity to explain the effects of predator–prey interactions (Almany 2004; Carroll et al. 2015; Hesterberg et al. 2017). Also, the density and size of mussels may be critical in off-setting the effects of one or more predator species each able to access and exploit different components of the mussel population (Garner and Litvaitis 2013). These findings have important consequences for ecological engineering projects (Firth et al. 2016) and the management of structures when the aim is to aid the reintroduction of species (e.g. canopy algae, Susini et al. 2007; Perkol-Finkel et al. 2012; or native oysters, Strain et al. 2017) while keeping in mind the role of biotic interactions (Ferrario et al. 2016; Gianni et al. 2018) to promote self-sustainability after initial restoration efforts.
